# Hydrogel Formulations for Topical Insulin Application: Preparation, Characterization and In Vitro Permeation across the Strat-M^®^ Membrane

**DOI:** 10.3390/polym15173639

**Published:** 2023-09-04

**Authors:** Aneta Ostróżka-Cieślik, Sławomir Wilczyński, Barbara Dolińska

**Affiliations:** 1 Department of Pharmaceutical Technology, Faculty of Pharmaceutical Sciences in Sosnowiec, Medical University of Silesia, Kasztanowa Street 3, 41-200 Sosnowiec, Poland; bdolinska@sum.edu.pl; 2 Department of Basic Biomedical Science, Faculty of Pharmaceutical Sciences in Sosnowiec, Medical University of Silesia, Kasztanowa Street 3, 41-200 Sosnowiec, Poland; swilczynski@sum.edu.pl

**Keywords:** hydrogel, insulin, Sepineo™ P 600, Sepineo™ PHD 100, Strat-M^®^ membrane, in vitro drug release study, rheology, texture analysis

## Abstract

Insulin has shown efficacy in the treatment of hard-to-heal wounds, which is mainly due to its role in regulating oxidative stress and inflammatory reactions. The aim of this study was to develop an insulin–hydrogel carrier based on Sepineo™ P 600 and Sepineo™ PHD 100 for application to lesional skin. Preformulation studies of the developed formulations were performed in terms of analysis of the pharmaceutical availability of insulin from the hydrogels through the Strat-M^®^ membrane, and rheological and texture measurements. Insulin is released in a prolonged manner; after a time of 6.5 h, 4.01 IU/cm^2^ (53.36%) and 3.69 IU/cm^2^ (47.4%) of the hormone were released from the hydrogel based on Sepineo™ P 600 and Sepineo™ PHD 100, respectively. Rheological analysis showed that the hydrogels tested belong to non-Newtonian, shear-thinning systems with yield stress. The insulin–hydrogel based on Sepineo™ P 600 and Sepineo™ PHD 100 shows optimal application properties. The results obtained provide a basis for further preclinical and clinical studies.

## 1. Introduction

At the beginning of the 20th century, there was a growing interest in insulin as a potential active substance to accelerate the regeneration of damaged skin. In our previous article, we reviewed studies published between 2000 and 2022 on the therapeutic efficacy of a hydrogel form of insulin for skin application [1]. The analysis confirms the efficacy of this hormone in the treatment of hard-to-heal wounds without causing side effects such as hypoglycemia. This is due to the fact that during tissue remodeling associated with the wound healing process, there is an increase in the expression of metalloproteinases, which degrades insulin in the skin of diabetics [2]. The topical application of insulin can reverse the impairment of diabetic wound regeneration processes and promote wound healing. The therapeutic effect of insulin after topical administration is mainly due to its role in regulating oxidative stress and inflammatory responses [1,2,3,4]. Chen et al. [5] found that insulin is involved in the early recruitment of neutrophils, increase in F2 macrophages and IL-10 levels [6]. Li et al. [7] conducted studies in a streptozotocin (STZ)-induced diabetic mice model. Topical application of insulin accelerates vessel maturation of wounds by regulating angiopoietin-1. Dhall et al. [8], in turn, confirmed that insulin reduces reactive oxygen species, which can lead to damaging proteins, lipids and DNA. Epidermal administration of the hormone stimulates keratinocyte migration and fibroblast response by the PI3K-AktRac1 pathway. Histological examination confirmed increased collagen levels in the tissue analyzed.

To our knowledge, there is no topical insulin application formulation on the market. A number of pharmaceutical forms of insulin for topical application are undergoing preclinical and clinical trials [1]. Research into the development of hydrogel forms of insulin seem particularly promising. Hydrogels are natural or synthetic polymer networks (or combinations of polymers) containing 30 to 90% water. Their undoubted advantage is their biocompatibility and impact on the wound healing process. The three-dimensional structure of the hydrogel provides a moist environment within the wound (clinically proven to accelerate the wound healing process), allows the formation of a protective barrier against wound infection, and enables the absorption of exudate by retaining it within its structure [9,10]. In addition, hydrogels show oxygen permeability, which promotes tissue regeneration and protects against the growth of anaerobic bacteria [11,12]. They are pleasant to apply and the technology for their manufacture is relatively simple and well understood. Hydrogel dressings promote keratinocyte differentiation and optimize the availability of growth factors and proteinases [13,14]. It was found that hydrogel carriers can exhibit bio-protective properties of the three-dimensional structure of therapeutic proteins against proteolytic and chemical degradation. Their porous structure and high-water content allow efficient accommodation of peptide-structured compounds in their matrix [15]. A significant disadvantage of hydrogels is their limited durability, which is due to their susceptibility to microbial contamination. In addition, they are characterized by low mechanical strength and susceptibility to degradation in vivo. An alternative to hydrogels is hydrocolloid dressings. These are usually produced as patches composed of an active colloid layer and a protective polyethylene or polyester outer layer.

Potential, previously unused polymers for the formulation of an insulin hydrogel carrier may be Sepineo™ P 600 (acrylamide/sodium acryloyldimethyl taurate copolymer/isohexadecane and Polysorbate 80) and Sepineo™ PHD 100 (Polyacrylate Crosspolymer-6). These are substances recommended for topical administration (in hydrogel or emulgel form). They have many advantages that predispose them to the development of dermatological drugs and cosmetics (e.g., easy to handle and incorporate into formulations, higher viscosity at lower concentrations, cold or hot formulation possible, good thermal stability, not require neutralization or rehydration steps or HLB calculation when developing hydrogels). Sepineo™ PHD 100 is an associative polymer combining polyelectrolytic repulsions and hydrophobic groups. It is stable in the pH = 2–8 range and compatible. The pH of the skin is 4.7–5.6. The physiological pH of the wound is <7.0 (slightly acidic) [16]. Sepineo™ P 600 used at low concentrations (0.5% and 5% (*w*/*w*) allows hydrogels with optimal viscosity to be obtained. It is characterized by good thermal stability, optimal rheological parameters (high viscosity at a low concentration) and facilitates the incorporation of both hydrophilic and hydrophobic substances into the structure of the polymer network [17,18,19,20].

The aim of this study was to develop an insulin–hydrogel carrier based on Sepineo™ P 600 and Sepineo™ PHD 100 for application to lesional skin.

## 2. Materials and Methods

### 2.1. Materials

Insulatard Penfil (INS) 100 IU/mL (human insulin, isophane, long-acting; excipients: zinc chloride, glycerol, metacresol, phenol, disodium phosphate dihydrate, sodium hydroxide, hydrochloric acid, protamine sulphate, water for injection) was purchased from Novo Nordisk (Bagsværd, Denmark). Sepineo™ P 600 and Sepineo™ PHD 100 were donated by Seppic (Paris, France). Glycerol 85% was purchased from Galfarm (Krakow, Poland). Phosphate-buffered saline (PBS) pH = 7.4 was purchased from Sigma-Aldrich Co. (St. Louis, MO, USA). All chemicals used in the study were of analytical grade. The Strat-M^®^ membrane was purchased from Merck Millipore (Burlington, MA, USA).

### 2.2. Preparation of Hydrogels

Hydrogels were prepared according to the manufacturer’s instructions in accordance with the principles of Good Manufacturing Practice (GMP) [21]. The water was heated to 70 °C before mixing. Sepineo™ P 600 and Sepineo™ PHD 100 were mixed with water by mechanical stirring (10 min at 500 rpm; Fisherbrand Isotemp stirring hotplate; Thermo Fisher Scientific, Mississauga, ON, Canada) until a hydrogel was obtained. The polymer matrix preparation process occurred in three steps: diffusion of water molecules into the polymer network (1), relaxation (loosening) of the polymer chains by hydration (2), and swelling of the polymer network (3) [22]. After 24 h, insulin at a dose of 1 mg/g (28.57 IU/g) was introduced into the resulting substrates using a mechanical mixing method. The composition of the formulation is shown in Table 1.

### 2.3. Stability Studies

The stability of the insulin hydrogels was tested based on the ‘Stability testing of biotechnological/biological products’ guidelines in compliance with the International Conference on Harmonization (ICH), Q5C. Samples of the formulations were stored at temperatures of 25 ± 1 °C and 5 ± 3 °C for a period of 4 weeks [23]. Testing included visual assessment, insulin content, pH, and rheological analysis in a plate-to-plate measurement geometry. The SevenCompact^TM^ S210 device (Mettler Toledo, Greifensee, Switzerland) was used to measure pH using the InLab^®^Expert Pro-ISM electrode with a solid polymeric electrolyte (Mettler Toledo, Switzerland). Over the range of pH values measured, the limiting error was ±0.01 pH. Color analysis and the hydrogel’s homogeneity were tested in glass beakers against two frosted screens, white and black [24]. Hydrogel resistance testing was determined by a centrifuge test: 4000 rpm for 30 min in a microcentrifuge (Model MPW-300, MPW Med. Instruments, Warsaw, Poland). The stability of the preparations under centrifugal force was assessed. The insulin content of the samples collected for analysis was determined by spectrophotometry using a UV/VIS CECIL spectrophotometer (CE 3021, Cambridge, UK) at λ = 271 nm. Dynamic viscosity was determined using a Lamy RM 200 Touch laboratory rotational viscometer (Lamy Rheology Instruments, Champagne au Mont d’Or, France) with a CP-1 Plus thermostatic system. The test used the MK-CP 2445 measuring system (diameter 24 mm, 0.45° angle). The above tests confirmed the stability of the hydrogels. The insulin content was within the acceptable limit, i.e., 90% of the initial value [25].

### 2.4. Insulin Release In Vitro

The diffusion capacity of insulin from the hydrogel substrate to the acceptor fluid was assessed in an Erweka DT600 paddle apparatus (Husenstamm, Germany) using the Enhancer Cell™ with a surface area of 3.80 cm^2^ (Erweka, Husenstamm, Germany). The extraction chambers were filled with 1g insulin hydrogel, a Strat-M^®^ membrane was applied and immersed in a beaker (200 mL) with the paddle. PBS acceptor fluid of 50 mL was used with sink conditions. The release test was conducted at 32 ± 1 °C (human skin surface temperature). The stirrer speed was 100 rpm. The amount of insulin released from the hydrogel was determined spectrophotometrically using a CECIL apparatus (CE 3021, Cambridge, UK). The analytical wavelength for API determination was determined: λ = 271 nm [26]. The linear dependence of absorbance as a function of the concentration of standard solutions was described by the equation y = 0.453x + 0.0072. The coefficient of determination was R^2^ = 0.999. The determined parameters: standard deviation, relative standard deviation of the results and confidence interval of the mean value indicate good accuracy and precision of the method. API release kinetics were analyzed using DDSolver 1.0 software (an addon for Microsoft Excel 2019), an addon for Microsoft Excel. The fit of the mathematical model (Zero Order, First Order, Higuchi, Korsmeyer–Peppas, Peppas–Sahlin, Hixson–Crowell, Hopfenberg, Baker–Lonsdale) to the insulin release data from the hydrogels was analyzed using adjusted R^2^ (correlation coefficient). The DDSolver1 software application was also used to compare release profiles [27,28].

The Strat-M^®^ membrane was used in the study. This is a synthetic membrane that offers a good alternative to studies using human and zoonotic skin [29]. The membrane is designed to mimic the structural and chemical properties of human skin, bypassing the biological processes occurring in living cells. The Strat-M^®^ membrane consists of a top layer with a lipid matrix resembling the human stratum corneum. This is followed by twin layers of PES, i.e., polyethersulfone (resembling dermis) and PO (polyolefin) non-woven fabric support (resembling subcutaneous fat tissue). Its thickness is 300 µm [30]. It was found to show a good correlation in permeability with the skin tissues. The Strat-M^®^ membrane is recommended for the evaluation of API permeation through the skin at the preclinical research stage [31,32,33,34,35,36].

### 2.5. Rheological Analysis

Rheological analysis was carried out in a plate-to-plate measurement geometry in rotation mode at 25 ± 1 °C (corresponding to ointment storage, package retrieval and application to human skin) and in the shear rate range of $\dot{\gamma }$ = 1–100 s^−1^ over 15 min. The application of the sample to the measurement system usually disturbs the structure and thermal equilibrium of the hydrogel, so it was left to rest for 30 min before the test was performed [28]. The tests were performed three times, each time using a fresh sample. A Lamy RM 200 touch rheometer was used (accuracy ± 1%, repeatability ± 0.2%). Analysis of the measurement data was carried out with the Rheometric-P Software. Flow curves were described with four mathematical models: Herschel–Bulkley Ostwald-de Waele, Bingham and Casson [37]:

(1) $$\mathrm{Ostwald}-\mathrm{de}\;\mathrm{Waele}:\;\;\;\;\;\;\;\;\;\;\;\tau =K\;\times \;{\dot{\gamma }}^{n},$$

(2) $$\;\;\;\;\mathrm{Herschel}–\mathrm{Bulkley}:\;\;\;\;\;\;\;\;\;\;\;\;\tau =\tau_{0}+K\;\times \;{\dot{\gamma }}^{n},$$

(3) $$\;\;\mathrm{Bingham}:\;\;\;\;\;\;\;\;\;\;\;\;\;\;\;\tau =\tau_{0}+\eta \;\times \;\dot{\gamma },$$

(4) $$\;\;\;\;\;\;\mathrm{Casson}:\;\;\;\;\;\;\;\;\;\;\;\;\;\;\;\;\;\tau^{0.5}={\tau_{0}}^{0.5}+\eta^{0.5}\;\times \;{\dot{\gamma }}^{0.5}$$

where:

τ, shear stress [Pa]; τ_0_, yield stress or yield point; $\dot{\gamma }$, shear rate [s^−1^]; K, consistency coefficient [Pa]^1/2^[s]^n^; η, viscosity [Pa × s];and n, flow behavior index.

Flow behavior studies. Flow curves were determined in the 40–250 Pa shear stress range. The measurement time was 15 min. 

Thixotropy studies. A hysteresis loop test was performed in the shear rate range $\dot{\gamma }$ = 1–100 s^−1^ (and backward) for 15 min. The area bounded by the hysteresis loop was calculated using the trapezoid method.

### 2.6. Texture Profile Analysis

Texture profile analysis (TPA) was performed using a Texture Analyzer TX-700 (Lamy Rheology, Champagne-au-Mont-d’Or, France). Preparation samples were tested using a ½ spherical probe with a diameter of 8 mm by compressing the sample twice (in 2 cycles). The head speed was 1 mm/s to a depth of 20 mm. The time between immersions was 20 s. The tests were carried out at 25 ± 1 °C. The box designed to hold the semi-solid form of the drug was filled with hydrogel. Thus, the preparation prepared was placed under the probe, which penetrated twice as deep into the sample. In the profile texture analysis, the following parameters were determined: hardness (maximum force recorded during the first compression cycle), elasticity (difference between the height of the sample before and after the first compression cycle), adhesiveness (work required to detach the probe from the hydrogel), and cohesiveness (ratio of work done in the direction of sample compression in the first and second cycles). The CRT (compression/relaxation/tension) test was performed with the same apparatus, using the following settings: down speed 0.5 mm/s; force to start 0.05 N; relaxation time 20 s, wait for position 20 mm. The tests were carried out at 25 ± 1 °C (corresponding to storage of the ointment, removal from the packaging and application to human skin). The relaxation parameter, i.e., the degree of relaxation of the cross-linked polymer as a result of penetrating the sample with the probe, was determined. A detailed presentation of the theoretical assumptions of texture analysis was discussed in a previous paper [28]. Rheotex software, version TX-UK01/2019, was used to automatically measure and analyze the results. The tests were performed three times, each time using a fresh sample.

### 2.7. Statistical Analysis

Results were presented as mean values ± standard deviation (SD). Data were analyzed with a two-tailed Student’s *t*-test, using Statistica 12.0 (Statsoft, Kraków, Poland). Data were considered statistically significant at *p* < 0.05. Statistically insignificant results were marked as NS.

## 3. Results

### 3.1. Stability Studies

The use of hydrogels as carriers for epidermally applied drugs protects the active substance molecule from unfavorable pH conditions, enzymes and allows the slow release of API.

Stability studies allow the suitability of formulations for therapy to be assessed. The absence of decomposition of the active substance is one of the key conditions for effective and safe use of the drug. Table 2 shows the results of the stability testing of the developed hydrogels. An acceptable change in hormone content was observed (in the case of low-stability substances, the amount of API should not change by more than 10%), which may be due to evaporation from the unit pack. 

### 3.2. Insulin Release In Vitro

**Figure 1:**
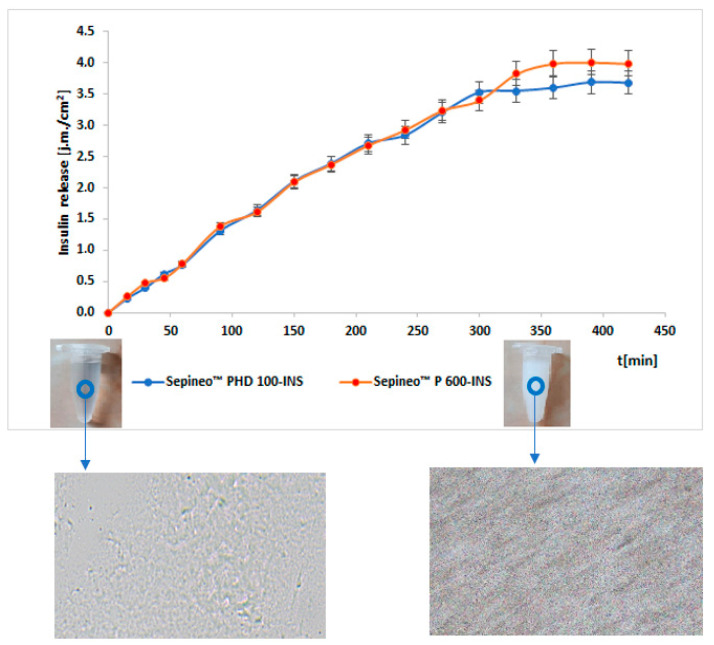
Insulin release profiles from hydrogels with microscopic images of preparations (100×; polarizing microscope LAB 40, Opta-Tech, Warsaw, Poland).

In vitro pharmaceutical availability studies indicate that insulin is released in a prolonged state due to the cross-linked hydrogel structure (Figure 1). After a time of 6.5 h, 4.01 IU/cm^2^ (53.36%) and 3.69 IU/cm^2^ (47.4%) of insulin were released from the Sepineo™ P 600 and Sepineo™ PHD 100-based hydrogel, respectively. After this time, it is likely that the concentration of the eroded polymer increased, which increased the viscosity of the system and, consequently, prevented further hormone release through the Strat-M^®^ membrane [38]. 

The similarity of the release profiles (Figure 1) was assessed using the difference (f1) and similarity (f2) factor method, as recommended by the European Medicines Agency (EMA) and U.S. Food and Drug Administration (FDA) regulations [39,40]. The analyzed insulin release profiles of Sepineo™ P 600 and Sepineo™ PHD 100 matrix were found to be similar. The parameter f1 was 4.39 (ranges from 0 to 15) with f2 being 99.78 (takes a value greater than 50 and close to 100).

Insulin release profiles from the developed hydrogels were analyzed using mathematical models comparing three parameters: R^2^ (coefficient of determination), AIC (Akaike information criterion), and MSC (model selection criterion) (Table 3). API release is assumed to follow a kinetic model, in which the parameters R^2^ and MSC take the highest values, while AIC takes the lowest values [41]. By analyzing the data in Table 1, it can be concluded that the release of hormone from the developed hydrogel matrices follows the Peppas–Sahlin model [42]. This model is characteristic of the kinetics of API release from hydrogels [42]. The release mechanism is based on diffusion of the active compound and relaxation of the polymer chains. The negative value of kPS1 (kPS1 = −0.220) determines the insignificant effect of Fick’s diffusion mechanism on insulin release compared to polymer chain relaxation (kPS2 = 0.121). The dominant mechanism is super case II transport brought on by relaxation in polymers and erosion [43,44,45]. It is presumably due to molecular electrostatic interaction during swelling and diffusion [46].

### 3.3. Rheological Analysis

**Figure 2:**
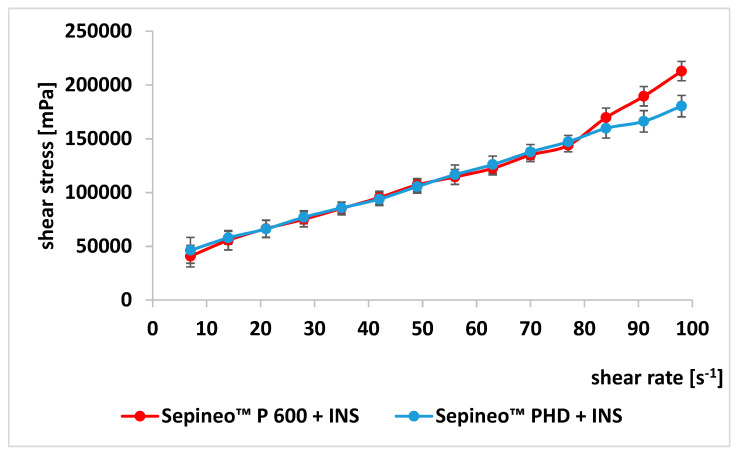
Flow rheograms of prepared formulations.

Knowledge of the rheological properties of hydrogels is important at the stage of their design, control, regulation of technological processes and acceptance of the preparation by the patient. Rheological parameters (viscosity, thixotropy) can significantly affect drug release and action [20]. The flow curves of both formulations are very similar (Figure 2). Table 4 shows the parameters for describing the flow curves with mathematical models. The Herschel–Bulkley model best describes the experimental flow curves, as evidenced by the higher R^2^ values. The hydrogel based on Sepineo™ P 600 with insulin shows a 33% lower yield stress (τ_0_, the force required to overcome Van der Waals-type cohesive forces and initiate flow) vs. Sepineo™ PHD 100 with insulin. The K-factor (which is a measure of fluid viscosity) of G1-INS is 14% lower vs. G2-INS. This is probably due to the larger particle size of Sepineo™ PHD 100. A parameter value of n < 1 indicates a pseudoplastic flow type (n = 1, Newtonian flow; n > 1, dilatant flow; n < 1, pseudoplastic flow) [47,48,49].

Based on the flow curves, it was found that the developed hydrogels with insulin were characterized by non-Newtonian flow and were shear-thinning with a tendency towards yield stress (Figure 2, Table 5). Shear thinning occurs due to the destruction of the polymer crosslink. The rate of breaking of intermolecular bonds during shearing of the sample while increasing the shear rate exceeds the rate of re-formation and consequently results in lower viscosity values [50]. Karavana et al. [51] found that formulations composed of entangled long-chain polymer molecules in a relaxed state under shear stress disentangle and release solvent located in molecular coils. As a consequence, the apparent viscosity decreases. An indicator of viscosity is thixotropy. Rheological analysis of hydrogels showed that they have thixotropic properties. A measure of thixotropy is the surface area bounded by curves (an ascending curve—with increasing shear rates and a descending curve—with decreasing shear rates). The small surface areas of the hysteresis loops indicate optimal recovery of the hydrogel structure [28,52]. The value of the area between the curves is G1-INS: 4764.8 Pa*s^−1^, G2-INS: 6888.7 Pa*s^−1^.

### 3.4. Texture Profile Analysis

**Figure 3:**
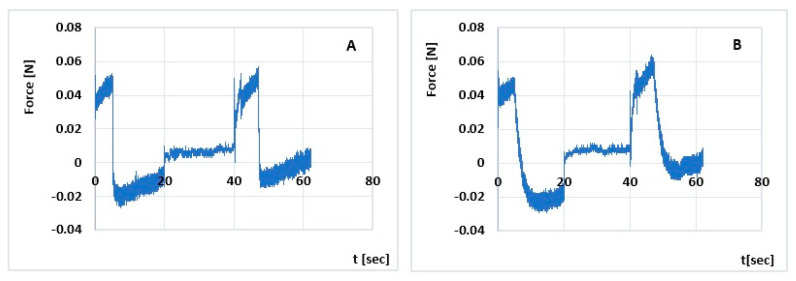
Texture profile analysis (TPA) of Sepineo™ P 600 with insulin (**A**), Sepineo™ PHD 100 with insulin (**B**).

**Figure 4:**
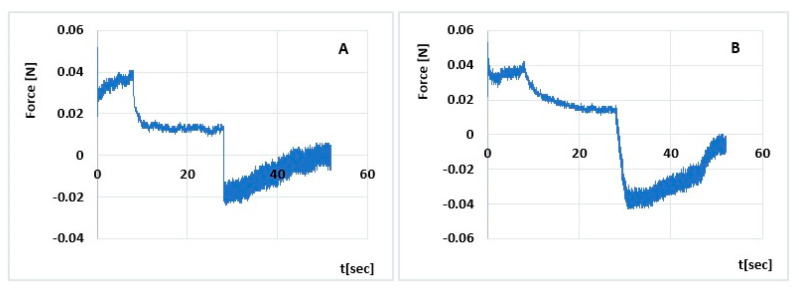
Penetration test (compression/relaxation/tension, CRT) of Sepineo™ P 600 with insulin (**A**), Sepineo™ PHD 100 with insulin (**B**).

Semi-solid preparations for topical administration should easily squeeze out of the unit package, be easily applied to the skin and remain in the wound bed without running off. The developed hydrogels should therefore be characterized by appropriate mechanical parameters, i.e., hardness, elasticity, adhesiveness and cohesiveness. Their analysis makes it possible to control the mechanical resistance to stress and, consequently, to assess their quality under conditions of use (squeezing out of the packaging, spreading on the skin surface). The results of the analyses are presented in Table 6 and Figure 3 and Figure 4.

The hardness test allows the force required to deform the hydrogel to be determined. Both formulations have a lower desirable hardness for easy application [49]. Sepineo™ PHD 100 with insulin shows a higher hardness than Sepineo™ P 600 with insulin by 4% (Hardness 1; NS) and 12% (Hardness 2; *p* < 0.05), respectively. This indicates a more compact matrix of the G2-INS formulation [53,54,55]. Hardness increases with increasing viscosity of the hydrogel, which was also confirmed by Ozcan [49]. Sepineo™ PHD 100 also has a 9% higher sample strain strength (cohesiveness G2-INS vs. G1-INS is 1.671 and 1.579, respectively; *p* < 0.05). Higher ‘cohesiveness’ values indicate structural recovery following hydrogel application [54]. Deformation/load tests alter the pore water content present between polymer chains. Saurez et al. [56] suggest that a more cohesive hydrogel binds water more strongly in its structure. Cohesiveness influences the more effective action of the formulation at the application site [49]. The G2-INS hydrogel also showed a 1.5 times (*p* < 0.05) greater ability to adhere (cohesiveness) to the packaging and interact with surrounding tissues. Elasticity, on the other hand, is defined as the direction of remodeling of a hydrogel after it is deformed by compression over a fixed time [53]. The higher value of the ‘elasticity’ parameter of the G2-INS formulation: 0.716 in relation to G1-INS: 0.693 (*p* < 0.05) indicates its lower elasticity. The preparations studied are characterized by distinct temporal relaxation responses. This is probably due to the difference in their porosity, which characterizes the hydrogel structure [57]. The polymeric bonds in a spatially cross-linked hydrogel form an interconnected porous structure that is filled with water when fully saturated. The porous structure is dependent on the type and concentration of formulation components, hydrogel maturation time and cross-linking density [58]. The observed differences in the magnitude of the analyzed mechanical parameters of the tested formulations do not negate the functional properties of either formulation. Both formulations show good sensory properties. They will spread easily on the skin and be removed from the unit pack.

## 4. Discussion

Effective insulin therapy of wounds depends largely on the type of substrate used. The correct choice of polymer influences the optimal contact of the preparation with the skin and the release of the hormone over a sufficiently long period of time. It is crucial that the polymer matrix is non-toxic, biodegradable, well tolerated by the patient and does not interact with insulin [8]. In turn, the formulation should be readily available to the patient and inexpensive. Based on these constraints, we are conducting research to develop a hydrogel carrier for insulin. Our previous research project was dedicated to the preformulation analysis of hydrogels based on Carbopol^®^ Ultrez^TM^ 10, Carbopol^®^ Ultrez^TM^ 30, methyl cellulose and glycerol ointment with insulin [28]. Here, we proposed two new hydrogel formulations with insulin.

The Sepineo™ P 600-INS system was characterized by a matt and milky color, while the Sepineo™ PHD 100-INS formulation was transparent. The formulations stored at temperatures: 25 ± 1 °C and 5 ± 3 °C for 4 weeks showed the required quality at both temperatures. No physical changes were observed to indicate reduced stability (Table 2). The structure of insulin was stable at the stage of incorporation into the polymer matrix, as demonstrated in our previous study by circular dichroism [28]. The lack of effect of homogenization on changes in the secondary structure of insulin was also confirmed by Quitério’s team, who prepared insulin-loaded poly-DL-lactide/glycolide (PLGA) nanoparticles [59]. The stability of the Sepineo™ P 600-based hydrogel is further enhanced by the presence of Polysorbate 80 in the formulation [17,60]. The formulations that developed had a suitable consistency and homogeneous appearance throughout the volume. The introduction of insulin into the hydrogels increased the pH of the substrate (pH_Sepineo™ PHD 100-INS_ = 7.16; pH_Sepineo™ P 600-INS_ = 7.14), which was within the skin-tolerable and non-irritating range, i.e., pH 4.0–10.0 [9,61,62].

Rheological analysis showed that the hydrogels studied belong to non-Newtonian, shear-thinning fluids with a yield stress, i.e., a limit to the force required to initiate the flow process [20,63]. The formulations show typical hydrogel characteristics with weak polymer–polymer interactions, which is an advantage for dermal application [20]. The value of the yield stress correlates with the application properties of the preparation (spreadability on the skin) and allows selection of optimal parameters for technological processes (mixing, filling of unit packs). The obtained flow curves (Figure 2) showed the best fit to the Herschel–Bulkley theoretical model. The curves are monotonic, without shear banding [64]. Texture profile analysis confirmed the hydrogel properties derived from rheological studies. The higher hardness and cohesiveness values were shown by the Sepineo™ PHD 100-INS formulation.

In our previous study [28], we measured the rheological properties of hydrogels with insulin based on Carbopol^®^ Ultrez^TM^ 10, Carbopol^®^ Ultrez^TM^ 30, methylcellulose, and glycerol ointment. Analysis of the rheograms showed that they have a course typical of shear-thinning non-Newtonian fluids with a flow limit. Analysis of the hydrogels at lower shear rates confirmed their highly dynamic viscosity: a characteristic of pseudoplastic liquids. In addition, the hydrogels that were studied exhibited a thixotropy effect characterized by a hysteresis loop. An intranasal hydrogel with insulin based on hydroxyethyl cellulose, which was developed by Von Zuben et al. [65], had similar rheological properties. The hydrogel was classified as a pseudoplastic (shear thinning) liquid (n < 1). The authors observed a decrease in the viscosity of the formulation with an increase in shear rate. They concluded that an increase in shear rate can cause thinning of the flow, which promotes a decrease in intermolecular interactions. They identified this feature as desirable for effective formulation application. After shear, the initial resistance for the formulation to flow decreases, which affects the ease of dosing. Agrawal et al. [66] developed an intranasal insulin hydrogel (Huminsulin^TM^) based on chitosan and polyvinyl alcohol (PVA). The formulation also had the characteristics of a pseudoplastic shear-thinning fluid, which is typical of most polymer-based hydrogels. The discussed features of semi-solid drug formulations allow the formation of a thin hydrogel layer at the site of application, with effective adhesion over an extended period of time.

The TPA profiles obtained in the study are typical for hydrogels for pharmaceutical applications. Three of the texture parameters analyzed are similar to those obtained in our earlier study [28] for insulin hydrogels based on Carbopol^®^ UltrezTM 30 (referred to as U30). Adhesiveness for G1-INS, G2-INS, and U30 are, respectively: 0.2 mJ, 0.3 mJ, and 0.3 mJ. Cohesiveness take the values: G1-INS: 1.579, G2-INS: 1.671, U30: 1.638, while elasticities are G1-INS: 0.693, G2-INS: 0.716, U30: 0.696. Differences were observed in hardness 1 (G1-INS: 0.053, G2-INS: 0.055, U30: 0.061) and hardness 2 measurements (G1-INS: 0.057, G2-INS: 0.064, U30:0.074). The results suggest a higher hardness of hydrogels with insulin based on Carbopol^®^ Ultrez^TM^ 30 by 15% and 11% (Hardness 1) and 30% and 16% (Hardness 2), respectively.

The higher viscosity of the Sepineo™ PHD 100-INS formulation (by 16% at η = 30 s^−1^) and, consequently, the stiffer matrix structure affected the lower amount of hormone released (47.4% vs. 53.36%) [67]. According to Huang et al. [68], the highly cross-linked polymer structure influences the slower release of API. The active substance also does not necessarily release completely. In an in vitro pharmaceutical availability study, prolonged release of insulin from the formulations developed was found, according to the Peppas–Sahlin kinetics model. Many authors have confirmed that drug release from a hydrogel matrix is related to the swelling rate of the carrier, which in turn depends on the physical structure of the polymer used [69,70,71]. The hydrogels tested exhibit a highly elastic structure. In addition, glycerol (a hydrophilic plasticizer) has the effect of reducing the swelling ratio by forming crosslinks between the polymer molecules. This limits the action of water as a drug diffusion channel [9]. In our earlier study [28], we showed that insulin release from hydrogel based on Carbopol^®^ Ultrez^TM^ 10 (H1-INS) and Carbopol^®^ UltrezTM 30 (H2-INS) (using cellulose dialysis membrane Spectra/Por^®^ 2/MWCO of 12–14 kDa) occurred according to the Higuchi model, while from hydrogel based on methylcellulose (H3-INS) and glycerol ointment (H4-INS) was according to the Korsmeyer–Peppas model. The hormone release profile, despite the use of one type of insulin (Actrapid, fast-acting insulin) was different. The H1-INS formulation released 70% of INS after 3 h, the H2-INS formulation released 65% also after 3 h, while the H3-INS and H4-INS formulations released after 9 h/75% of INS, after 6 h/60% of INS, respectively. Von Zuben et al. [65] proposed hydroxyethylcellulose as a base for developing a hydrogel formulation of insulin. They used Novolin R^®^, a short-acting insulin solution. The model membrane was a synthetic cellulose acetate membrane. After 4 h, 90.74% of INS was released, according to kinetics based on the Weibull model. Agrawal et al. [66] studied the release of insulin (Huminsulin^TM^) from chitosan (CS) and polyvinyl alcohol (PVA)-based hydrogel with a composition of 1% CS, 4% PVA; 2% CS, 3% PVA; 3% CS, 2% PVA; 4% CS, 1% PVA; 5% CS, 0% PVA. Through the egg membrane in vitro, 60–90% of the hormone was released within 6 h. The results obtained in four different studies suggest that the amount of insulin released and the rate at which this process occurs depends primarily on the properties of the hydrogel matrices used. The insulin release profile, on the other hand, presumably does not depend on the type of commercial insulin preparation used, which needs to be confirmed by additional testing.

It can be assumed that the affinity of the IRS (insulin receptor substrate) receptors for insulin allows the generation of cellular responses to already low concentrations of the hormone that will be released from the hydrogel. IRS-1 and IRS-2 mediate insulin action in the skin under physiological conditions, influencing the normal development and function of this organ [72]. A disadvantage of topical application of insulin is its short half-life (2–5 min) [73]. The prolonged release of insulin from the hydrogel overcomes this problem, while preventing the need for frequent application of the product [74].

Of note is the choice of membrane used in the in vitro study of insulin pharmaceutical availability from hydrogels, i.e., the Strat-M^®^ membrane. An analysis of the literature indicates a significant recent increase in its use in studies of API release from a semi-solid drug formulation [75,76,77]. Also, the European Medicines Agency recommends the use of synthetic membranes in drug release assays [78]. The Strat-M^®^ membrane was found to be an effective substitute for human and animal skin, without lot-to-lot variability. It mimics the multilayered structure of natural skin and avoids the ethical issues that arise from conducting animal testing in the preclinical research phase [79]. The Strat-M^®^ membrane can be used to permeate both hydrophilic and lipophilic compounds [77].

This study is part of the current trend to find safe and effective ways of topical application of the hormone. In perspective, the proposed insulin formulation can be prepared in the pharmacy prescription room for a specific patient with a dry wound (occlusive action) or a moist and oozing wound (absorption of excess fluid, cooling). More advanced materials/preparations for long-term delivery of bioactive insulin, e.g., transdermal drug delivery systems, nano-drug delivery systems, sponge dressing, and electrospun polymeric fibers are also under investigation. For example, Li et al. [80] conducted a study on silk fibroin dressing with insulin. Approximately 90.7% of the hormone was released from the formulation within 28 days. In a study by Ehterami et al. [81], insulin-delivering chitosan nanoparticles were coated onto the electrospun poly (ε-caprolactone)/Collagen. Overall, 64.36% of the insulin was released from the formulation within 14 days. It is important to bear in mind that one of the key challenges of developing insulin formulation technology is ensuring the sterility of the developed preparation. Insulin is sensitive to temperature, radiation, and pressure. Available sterilization methods affect the inactivation of this hormone and may result in a loss of viscosity of the hydrogel.

## 5. Conclusions

A limiting barrier to topical insulin therapy is the development of a safe carrier that will effectively deliver the hormone to the wound bed. Preformulation studies demonstrate that the formulations developed are stable and provide prolonged insulin release. The insulin hydrogels based on Sepineo™ P 600 and Sepineo™ PHD 100 show optimal application properties. Due to the presence of water, they can maintain a moist wound environment (which allows cell growth and migration), and thus stimulate healing processes and soften dry necrotic tissue. The results obtained provide a basis for further preclinical and clinical studies.

## Figures and Tables

**Table 1:** Composition of formulations.

	Sepineo™ P 600 Hydrogel G1 [%*w*/*w*]	Sepineo™ PHD 100 Hydrogel G2 [%*w*/*w*]
Sepineo™ P 600	4	-
Sepineo™ PHD	-	2
Glycerol 85%	3	3
Distilled water	Ad 100	Ad 100

**Table 2:** Results of the hydrogel stability test.

Conditions	Weeks	Color	Phase Separation	pH	Drug Content	Viscosity η (30 s^−1^) [mPa × s]	Photos of Hydrogels
G1-INS							
5 ± 3 °C	2	No color change	No	7.11 ± 0.05	99.5% ± 0.5	3940 ± 204	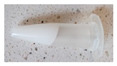
	4	No color change	No	7.12 ± 0.03	99.6% ± 0.5	3960 ± 114	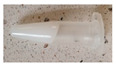
25 ± 1 °C	2	No color change	No	7.16 ± 0.05	99.7% ± 0.4	4080 ± 199	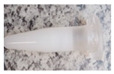
	4	No color change	No	7.15 ± 0.02	100.5% ± 0.2	4091 ± 200	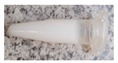
G2-INS							
5 ± 3 °C	2	No color change	No	7.16 ± 0.04	99.2% ± 0.4	4320 ± 180	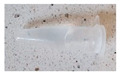
	4	No color change	No	7.11 ± 0.03	99.8% ± 0.3	4510 ± 211	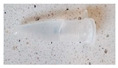
25 ± 1 °C	2	No color change	No	7.09 ± 0.04	99.8% ± 0.2	4680 ± 202	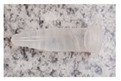
	4	No color change	No	7.11 ± 0.01	101.2% ± 0.5	4820 ± 200	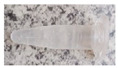

**Table 3:** Mathematical models describing the kinetics of insulin release from hydrogels.

Kinetics Models	Equation	Hydrogel	Parameters	R^2^_adjusted_	AIC	MSC
Zero Order model	f = k_0_ × t	G1-INS G2-INS	k_0_ = 0.012 k_0_ = 0.011	0.9790 0.9369	−9.0372 7.1041	3.7198 2.6294
First Order model	f = 100 × [1 − e^−k1×t^]	G1-INS G2-INS	k_1_ = 0.001 k_1_ = 0.001	0.9813 0.9412	−10.6466 6.0386	3.8347 2.7004
Higuchi model	f = k_H_ × t^0.5^	G1-INS G2-INS	k_H_ = 0.184 k_H_ = 0.181	0.8933 0.9123	13.7068 12.0342	2.0952 2.3007
Korsmeyer-Peppas model	f = k_KP_ × t^n^	G1-INS	k_KP_ = 0.030	0.9946	−27.2065	5.0176
			n = 0.834			
		G2-INS	k_KP_ = 0.046			
			n = 0.749	0.9796	−8.9155	3.6973
Peppas-Sahlin model	f = k_PS1_ × t^m^ + k_PS2_ × t^(2×m)^	G1-INS	k_PS1_ = −0.220	0.9961	−30.7982	5.2741
			k_PS2_ =0.121			
			m = 0.324			
		G2-INS	k_PS1_ = −0.973	0.9882	−16.3418	4.1924
			k_PS2_ = 0.561			
			m = 0.217			
Hixson-Crowell model	f = 100 × [1 − (1 − k_HC_ × t)^3^]	G1-INS G2-INS	k_HC_ = 0.0 k_HC_ = 0.0	0.9805 0.9398	−10.1033 6.3962	3.7959 2.6766
Hopfenberg model	f = 100 × [1 − (1 − k_HB_ × t)^n^]	G1-INS	k_HB_ = 0.0	0.9797	−8.6180	3.6869
			n = 58.345			
		G2-INS	k_HB_ = 0.0	0.9366	8.0568	2.5659
			n = 58.939			
Baker–Lonsdale model	3/2 × [1−(1 − F/100)^2/3^] − F/100 = k_BL_ × t	G1-INS G2-INS	k_BL_ = 0.0 k_BL_ = 0.0	0.8915 0.9108	13.9423 12.2931	2.0784 2.2834

Abbreviations: G1, Sepineo™ P 600 with insulin; G2, Sepineo™ PHD with insulin; f, amount of the drug release; t, time; k_0_, reaction rate coefficient; k_1_, rate constant; k_H_, dissolution constant; k_KP_, constant depicting the experimental parameters based on geometry and dosage forms; n, release exponent; k_PS1_, Peppas–Sahlin release constant (constant for Fickian diffusion); k_PS2_, constant for Case II relaxational mechanism; m, diffusion exponent; k_HC_, Hixson–Crowell release constant; k_HB_, Hopfenberg release constant; k_BL_, Baker Lonsdale release constant, R^2^_adjusted_, adjusted correlation coefficient, AIC, Akaike Information Criterion; MSC, Model Selection Criteria.

**Table 4:** The results obtained from the mathematical modeling of rheogram.

Formula Code	Herschel–Bulkley				Ostwald-de Waele			Bingham		Casson	
	τ_0_	n	K	R^2^	n	K	R^2^	τ_0_	R^2^	τ_0_	R^2^
G1-INS	32200	0.993	2.84	0.912	0.930	3.76	0.760	74282	0.639	4701	0.688
G2-INS	48000	0.945	3.30	0.983	0.538	15.5	0.901	33006	0.880	15712	0.905

Symbols: τ_0_, the Yield stress [mPa]; K, the consistency index [Pa*s^n^]; n, the flow behavior index; R^2^, regression coefficient.

**Table 5:** Apparent viscosity values at different shear rates (Mean ± SD, n = 3).

Hydrogel	η (30 s^−1^) [mPa × s]	η (50 s^−1^) [mPa × s]	η (100 s^−1^) [mPa × s]
G1-INS	3241 ± 50	1949 ± 91	1387 ± 71
G2-INS	3772 ± 87	2070 ± 101	1462 ± 113

**Table 6:** Mechanical parameters of hydrogels (Mean ± SD, n = 3).

Formula Code	Relaxation [%]	Hardness 1 [N]	Hardness 2 [N]	Cohesiveness	Adhesiveness [mJ]	Elasticity
G1-INS	76.4% ± 1.06	0.053 ± 0.01	0.057 ± 0.03	1.579 ± 0.05	0.2 ± 0.02	0.693 ± 0.07
G2-INS	71.5% ± 0.62	0.055 ± 0.02	0.064 ± 0.01	1.671 ± 0.06	0.3 ± 0.01	0.716 ± 0.05
*p*	<0.05	NS	<0.05	<0.05	<0.05	<0.05

## Data Availability

The data presented in this study are available upon request from the corresponding authors. The donators had no role in the design of the study; in the collection, analyses, or interpretation of data; in the writing of the manuscript; or in the decision to publish the results.
